# A Nitrate-Transforming Bacterial Community Dominates in the *Miscanthus* Rhizosphere on Nitrogen-Deficient Volcanic Deposits of Miyake-jima

**DOI:** 10.3390/microorganisms11020260

**Published:** 2023-01-19

**Authors:** Ahmad Arsyadi, Yong Guo, Akiko Ebihara, Nobuo Sakagami, Midori Sakoda, Kanako Tago, Takashi Kamijo, Hiroyuki Ohta, Tomoyasu Nishizawa

**Affiliations:** 1 Graduate School of Agriculture, Ibaraki University, Ibaraki 300-0393, Japan; 2 College of Agriculture, Ibaraki University, Ibaraki 300-0393, Japan; 3 United Graduate School of Agricultural Science, Tokyo University of Agriculture and Technology, Tokyo 183-8509, Japan; 4 Institute for Agro-Environmental Sciences, National Agriculture and Food Research Organization (NARO), Ibaraki 305-8604, Japan; 5 Faculty of Life and Environmental Sciences, University of Tsukuba, Ibaraki 305-8572, Japan

**Keywords:** nitrate-reducing bacteria, metagenome, pioneer plant, volcanic deposits, rhizosphere

## Abstract

The perennial gramineous grass *Miscanthus condensatus* functions as a major pioneer plant in colonizing acidic volcanic deposits on Miyake-jima, Japan, despite a lack of nitrogen nutrients. The nitrogen cycle in the rhizosphere is important for the vigorous growth of *M. condensatus* in this unfavorable environment. In the present study, we identified the nitrogen-cycling bacterial community in the *M. condensatus* rhizosphere on these volcanic deposits using a combination of metagenomics and culture-based analyses. Our results showed a large number of functional genes related to denitrification and dissimilatory nitrate reduction to ammonium (DNRA) in the rhizosphere, indicating that nitrate-transforming bacteria dominated the rhizosphere biome. Furthermore, nitrite reductase genes (i.e., *nirK* and *nirS*) related to the denitrification and those genes related to DNRA (i.e., *nirB* and *nrfA*) were mainly annotated to the classes Alpha-proteobacteria, Beta-proteobacteria, and Gamma-proteobacteria. A total of 304 nitrate-succinate-stimulated isolates were obtained from the *M. condensatus* rhizosphere and were classified into 34 operational taxonomic units according to amplified 16S rRNA gene restriction fragment pattern analysis. Additionally, two strains belonging to the genus *Cupriavidus* in the class Beta-proteobacteria showed a high in vitro denitrifying activity; however, metagenomic results indicated that the DNRA-related rhizobacteria appeared to take a major role in the nitrogen cycle of the *M. condensatus* rhizosphere in recent Miyake-jima volcanic deposits. This study elucidates the association between the *Miscanthus* rhizosphere and the nitrate-reducing bacterial community on newly placed volcanic deposits, which furthers our understanding of the transformation of nitrogen nutrition involved in the early development of vegetation.

## 1. Introduction

The soil–plant ecosystem is the main reservoir of elements such as carbon and nitrogen, and the main players in the element/material cycle are plants and microorganisms. Although carbon and nitrogen are not contained in the soil *parent materials*, these elements are introduced by physical deposition from the atmosphere and biological fixation. Primary succession on glacier forelands [1] and volcanoes [2] presents an ideal opportunity to understand the formation of terrestrial ecosystems through biological colonization.

Miyake Island (Miyake-jima) in Japan is an active volcanic island located on the western rim of the Pacific Ocean, with a humid subtropical climate. Since 1084, there have been fifteen eruptions on Miyake-jima, with the most recent eruptions occurring in 1874, 1940, 1962, 1983, and July–August 2000 [3]. The effects on the ecosystem of the last eruption involved the formation of a new caldera and the deposition of ash to form acidic volcanic deposits (pH 3–4) that lack carbon and nitrogen but have high amounts of either exchangeable cation calcium or aluminum. The area around the crater was exposed to poisonous gases such as sulfur dioxide and hydrogen sulfide, which caused extensive damage to the vegetation in the area. After the eruption in 2000, almost all vegetation that covered around the crater was lost.

Sato et al., (2009) clarified that pioneer microorganisms of volcanic ash deposits (pH 3) acidified with volcanic gas are chemotrophs near the mountaintop of Oyama, Miyake-jima [4]. Fujimura et al., (2012 and 2016) revealed the role of early microbial communities in supporting pioneering plant colonization in unvegetated volcanic deposits, suggesting that the bacterial community structure transitioned from chemotrophs (autotrophs) to heterotrophs [2,5]. In addition, Guo et al. (2014) and Lathifah et al. (2019) reported that successional change in the early soil microbial community occurred with primary vegetation that developed on the newly placed volcanic deposits, in which the plant benefit groups are mainly affiliated with the orders Burkholderiales and Rhizobiales, which are significantly increased with the vegetation succession [6,7]. Guo et al. (2021) further revealed that different plant benefit groups dominated the rhizosphere in different developmental phases on the recent Miyake-jima volcanic deposits, i.e., *Paraburkholderia* and *Trinickia* represented the major plant-beneficial groups in the early colonization phase, whereas Rhizobiales and *Arthrobacter* increased in the later colonization phase [8]. *Miscanthus* grass is considered tolerant to high Al^3+^ concentrations and acidic conditions, as well as nitrogen deficiency [9], whereas associated arbuscular mycorrhizal fungi have been reported to support *Miscanthus* colonization on acidic soils [10,11]. In addition to the dominance of nitrogen-fixing bacteria, a considerable proportion of denitrification-related genes (i.e., *nirK*, *nirS*, *norB*, and *nosZ*) were detected as increasing in the 3.5–9.5-year-old Miyake-jima volcanic deposits at an unvegetated study site [2]. However, those genes associated with a competitive process in soils, i.e., dissimilatory nitrate reduction to ammonium (DNRA), have not yet been focused.

Denitrification and DNRA are thought of as the major microbial processes of nitrate transformation in terrestrial ecosystems [12]. The former induces gaseous losses of nitrogen from soils via the reduction of nitrate (NO_3_^−^) to nitrous oxide (N_2_O) and/or dinitrogen (N_2_) [13,14,15], whereas the latter reduces NO_3_^−^ to ammonium (NH_4_^+^), protecting NO_3_^−^ from leaching and gaseous losses [16] and enriches soils with readily available NH_4_^+^ for plants, such as N-fertilizer [12]. Therefore, the investigation of denitrification- and DNRA-associated genes and communities in the rhizosphere is expected to elucidate the supply of N-nutrition to pioneer plants and gain a comprehensive understanding of vegetation recovery on barren land.

Recent advances in high-throughput sequencing technologies revealed that nitrogen-cycling microbes are unexpectedly diverse in their functions and phylogenies [17]. Vegetation recovery after the volcanic eruption on Miyake-jima presents an ideal opportunity to increase understanding of the formation of the terrestrial ecosystem. However, there is still lack of information on the nitrogen-cycling microbial community associated with the pioneer plant *Miscanthus*, particularly, those associated with the processes of denitrification and DNRA. The objective of this study was to characterize and identify the nitrogen-cycling bacterial community associated with the *Miscanthus* rhizosphere on Miyake-jima volcanic deposits based on shotgun metagenomic and culture-based analyses.

## 2. Materials and Methods

### 2.1. Site Description and Sample Collection

**Figure 1:**
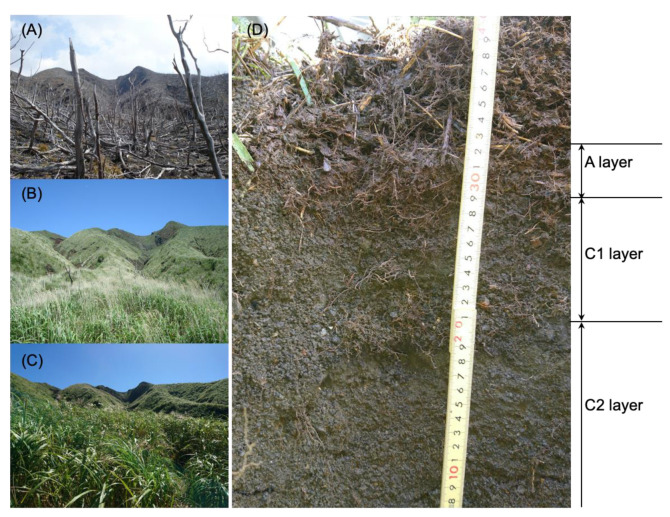
Vegetation damage and colonization of *Miscanthus* at site IG7. (**A**) March 2008; (**B**) May 2016; (**C**) September 2018; (**D**) soil profile in September 2018. A layer of bulk soil with the *Miscanthus* rhizosphere (0–11 cm); C1 layer, upper year 2000-volcanic deposits (11–22 cm); C2 layer, lower year 2000-volcanic deposits (>22 cm).

Miyake-jima, designated as one of national parks of Japan (https://www.kankyo.metro.tokyo.lg.jp/naturepark/english/know/park/introduction/kokuritsu/fujihakone/miyakejima/index.html, accessed on 24 November 2022), provides a natural ecological model to study the ecosystem restoration after the volcanic eruption. We were authorized to collect the Miyake-jima soil, volcanic deposit, and plant samples as the minimum required by this investigation; therefore, we collected one replicate of each sample for the sampling events in May 2016, September 2017, and September 2018. Site IG7 was established on the windward (north) side of Mt. Oyama (34°05.37′ N and 139°30.84′ E) of Miyake-jima at an altitude of 547 m a.s.l. (Figure 1). The climate type in this region is humid subtropical, with a mean annual rainfall of 3024.7 mm and a mean air temperature of 18.0 °C from 1991 to 2020, according to Japan Meteorological Agency (https://www.data.jma.go.jp/obd/stats/etrn/view/nml_sfc_ym.php?prec_no=44&block_no=47677&year=&month=&day=&view=, accessed on 24 November 2022). Vegetation has gradually recovered halfway up the mountaintop since 2001 with the colonization of *Miscanthus condensatus*, belonging to the family *Poaceae* as one of the pioneer grasses (Figure 1A–C) [18,19,20,21]. At site IG7, the surface coverage of *M. condensatus* increased as follows: <20% in 2009, <45% in 2013, and 90% in 2018. Complete *Miscanthus* grass samples (approximately 70 cm in height and 12 cm width at the root rhizosphere) were collected in September 2017 and September 2018. In March 2016, only volcanic deposit layer (referred to as the C layer), in 2017, a new organic soil layer (referred to as the A layer) on surface and C layer (>20 cm in depth), and in 2018, the A layer (0–10 cm in depth) and the upper (10–20 cm in depth) and lower (20–30 cm in depth) volcanic deposit layers (referred to as the C1 and C2 layer, respectively) were observed and randomly sampled from several plots and mixed in sterile plastic bags. All samples were kept at 4 °C and transported to laboratory within 2 days.

### 2.2. Soil Chemical Properties Analysis

A portion of C, C1, C2, and A samples (4–8 g in weight) was dried at 105 °C for over 12 h. Total carbon and total nitrogen in the dried samples were determined using a JM3000 CN counter with a JMA 3000 Auto Samper (J-Science Lab., Kyoto, Japan). The volumetric water content was calculated as reported previously [6]. A slurry consisting of a 1:2.5 mass ratio of wet sample and distilled water was used to determine the sample pH value. Fresh C1, C2, and A samples (5–15 g in weight) were used to determine nitrate (NO_3_^−^) and ammonium (NH_4_^+^) contents using an SPCA-6210 (Shimadzu, Kyoto, Japan). The measurement was initiated by 1 N KCl extract and mixed solution sample with either distilled water for NO_3_^−^ or a combination of solution A (phenol, 20% NaOH/sodium hydroxide, acetone) and solution B (NaClO/sodium hypochlorite) for NH_4_^+^. All analyses were performed in triplicates except for water content measurements.

### 2.3. Sample Pretreatment of Miscanthus

The root-rhizosphere of *Miscanthus* grass at the IG7 site, which was sampled in 2017 and 2018, was subjected to pretreatment for metagenomic and culture-dependent analyses. To obtain the rhizosphere soil, we used soil-associated roots (tiny soil) with consideration for the root growth features of *Miscanthus*, which forms a net-like structure and binds loose soil. Due to the one plant colony that was obtained for each sampling event, the rhizosphere soils were recovered from the plant roots as fully as possible and were pooled together to reduce bias. The *Miscanthus* roots were cut and separated using sterilized scissors in sterile conditions. Approximately 3 g of root sample was transferred to 30 mL of sterile distilled water in a 50 mL Falcon tube [8]. In order to leach the rhizosphere soil from roots, the samples were sonicated using an EYELA ultrasonic cleaner machine (Tokyo Rikakikai, Kanagawa, Japan) for about 5 min. This operation was repeated several times as necessary. The suspension of rhizosphere soil was centrifuged at 10,000× *g* for 5 min to form pellets. After removing the supernatant, the pellets were treated as rhizosphere samples. Several preparations of rhizosphere soil pellets were pooled together and stored at −20 °C until DNA extraction for shotgun metagenomic analysis. In addition, the main and lateral roots were separated as shown in Figure S1, and then the adhered soil was recovered in sterile distilled water in accordance with the above-mentioned method. The suspensions of rhizosphere soil recovered from the main and lateral roots were stored at 4°C and used for the bacterial isolation within 1 day.

### 2.4. Genome Sequencing, Assembly, and Annotation of Metagenomic Contigs

The extraction of total DNA from 0.75 g rhizosphere sample of *Miscanthus* was performed using a DNeasy *PowerSoil kit* (QIAGEN, Hilden, Germany) in accordance with the manufacturer’s instructions. The obtained DNA (16.2 μg in total) was used to build a paired-end library with insert size of ~550 bp sequenced on an Illumina HiSeq2500 platform at GeneBay, Yokohama, Japan (http://genebay.co.jp, accessed on 24 November 2022). The obtained reads were analyzed using a metagenomic data analyzing pipeline metaWRAP version 1.0 (https://github.com/bxlab/metaWRAP/releases/tag/v1.0, accessed on 24 November 2022) [22]. Briefly, the raw reads were trimmed using the metaWRAP Read_qc module with default parameters and were assembled using the metaWRAP Assembly module with the megahit option and default settings [23]. All the outputted contigs were used to identify their taxonomy using the metaWRAP Kraken module and to calculate their abundance in each sample using the metaWRAP Blobology module with default parameters. The ribosomal RNA genes and protein-coding sequences (CDSs) were predicted using Barrnap version 0.9 (https://github.com/tseemann/barrnap, accessed on 24 November 2022) and MetaGeneMark (http://exon.gatech.edu/meta_gmhmmp.cgi, accessed on 24 November 2022) [24], respectively. The gene function and taxonomic identification of the predicted CDSs was assigned using GhostKOALA version 2.2 with the KEGG database [25], and the COG categories were assigned using RPS-BLAST against the COG database [26]. To calculate the relative abundances of the CDSs, the reads outputted from the metaWRAP Read_qc module were mapped to contigs using the BWA version 0.7.17 options of “index” and “mem” with the default parameters, and then the resulting SAM file was converted to BAM format and sorted using the Samtools version 1.9 options of “view” and “sort” [27]. Finally, the relative abundances of the CDSs were calculated as gene copies per million mapped reads (CPM) using the tool “jgi_summarize_bam_contig_depths” of MetaBAT version 2.12.1 [28].

### 2.5. Isolation of the Miscanthus Root-Associated Bacteria

The soil rhizosphere suspension (1.0 mL) was transferred to 9.0 mL of sterile distilled water to make 10^−2^, 10^−3^, and 10^−4^ dilutions [6]. Then, 100 µL of each diluted soil suspension was spread onto 1.5% agar plates of 100-fold diluted nutrient broth medium supplemented with 3.0 mM sodium nitrate (NaNO_3_) and 4.4 mM sodium succinate (DNB-NS medium and pH 6.8) in triplicates. The plates were grown both aerobically and anaerobically at 30 °C for 2 weeks [29,30]. Additionally, to retard bacterial growth, DNB-NS medium supplemented with antibiotic nalidixic acid was also used in this study and grown anaerobically at 30 °C for 2 weeks [31]. Single colonies appearing on the 10^−3^ and 10^−4^ plates were selected according to unique morphotypes [32], and were subcultured repeatedly on the same media with the same incubation conditions in order to obtain pure cultures of isolates.

### 2.6. Terminal-Restriction Fragment Length Polymorphism (T-RFLP) Profiling

A portion of the 10^−2^ plates containing grown isolates as described above was used in this analysis. Total genomic DNA was extracted from the bacterial cells grown on the plates using ISOIL for beads beating kit (Nippon Gene, Tokyo, Japan) in accordance with the manufacturer’s procedures. Polymerase chain reaction (PCR) amplification of the bacterial 16S rRNA gene was performed as follows: the total volume of the PCR mixture was 30 µL, which contained 1 µL of template DNA (100 ng/µL), 0.5 µL of 10 µM forward primer (Q-10F, 5′-CAGTTTGATCCTGGCTCAG-3′; J-Bio21, Kisarazu, Japan), 0.5 µL of 10 µM reverse primer (926r, 5′-CCGTCAATTCCTTTRAGTTT-3′) [4], 3 µL of 10 × *Ex Taq* buffer (20 mM Mg^2+^ plus), 3 µL of dNTP mixture (2.5 mM each), and *TaKaRa Ex Taq* polymerase (TaKaRa Bio, Shiga, Japan). Q-10F primer labelled with a quenching fluorescence was purchased from J-Bio21. PCR conditions were initially 95 °C (2 min), then 25 cycles of 95 °C (30 s), 54 °C (45 s), and 72 °C (90 s). PCR reactions were carried out in a TaKaRa PCR Thermal Cycler Dice^®^ *Touch* (TaKaRa Bio). The PCR products were purified using a QIAquick PCR purification kit (QIAGEN) in accordance with the manufacturer’s protocol. *Alu*I, *Hae*III, *Hha*I, and *Msp*I (TaKaRa Bio) were used for restriction enzyme digestion, and the fragments were purified using a QIAquick PCR purification kit (QIAGEN) [33]. Two microliters of terminal-restriction fragments (T-RFs) were mixed with 12 µL of Hi-Di™ Formamide Genetic-analysis grade (Applied Biosystems, Foster City, CA, USA) and 0.1 µL of GeneScan™ 500 Liz^®^ Size Standard. T-RFs were denatured at 96 °C for 2 min and quenched thereafter. The length of T-RFs was determined using an ABI PRISM 3130*xl* Genetic Analyzer (Applied Biosystem) in GeneMapper mode [33]. Analysis for the determination of T-RF lengths was performed by using GeneMapper^®^ Software version 3.7 (Applied Biosystems, Foster City, CA, USA). T-RF data were developed using T-REX software [34]. T-RFs whose sizes were under 50 bases and <0.5% of the total area were omitted from this analysis. T-RFs with a different size that was less than 0.5 bp, digested with the same enzyme, and obtained from the same sample, were grouped as one T-RF. Based on the T-RFs profile, a Bray–Curtis-based distance matrix of the cultivated bacterial communities was calculated using the vegan package of R software version 3.5.3 and visualized using a wild bootstrapping dendrogram [35].

### 2.7. Amplified Ribosomal DNA Restriction Analysis (ARDRA)

Direct PCR of intact bacteria (colony PCR) method was used to amplify the 16S rRNA gene of each obtained strain (i.e., a pure culture) using the 10F and 1541R primer pair (10 µM) [4]. The reaction mixture (10 µL) contained 5 µL of GoTaq Green Master Mix (Promega, Madison, WI, USA), 0.5 µL of each primer, 4 µL of sterile milliQ water, and a selected bacterial colony. Prior to PCR, heat treatment was performed to lyse the cells using TaKaRa PCR Thermal Cycler Dice^®^ *Touch*, with the following conditions: denaturation at 95–96 °C for 8 min. Amplification of the bacterial 16S rRNA gene was carried out with the following conditions: initially 95–96 °C (8 min), followed by 25 cycles of 95 °C (30 s), 55 °C (60 s), and 72 °C (120 s). After the final extension at 72 °C for 7 min, the PCR mixtures were kept at 4 °C. The PCR products were digested directly with *Hin*fI (50 U/µL, TaKaRa Bio) by mixing 4.5 µL of PCR product with 1 µL of restriction enzyme. The reaction mixtures (5.5 µL) were incubated at 37 °C for 1 h and electrophoresed using 1.5% agarose LO3 (TaKaRa Bio) with TAE (Tris-Acetate-EDTA) buffer to visualize the restricted fragment (RF) pattern. Strains with an identical RF profile were grouped as an operational taxonomic unit (OTU) through manual assessment according to a previous report [36]. The relative abundance of each OTU was determined by dividing the OTU number by the total number of OTUs. The heatmap of OTU abundance in total was visualized based on log_2_ transformations of the OTU table using the formula log_2_(10 × *x* + 1), where *x* is the relative abundance of each OTU.

### 2.8. Identification of Representative Strains of Each OTU Based on the 16S rRNA Gene

Genomic DNA was extracted from the bacterial cells using a phenol-chloroform extraction method [4]. Amplification of the 16S rRNA gene was performed using the 10F and 1541R primer pair (10 µM) [4]. The total volume of the PCR mixture was 50 µL, which contained 1 µL of template DNA (100 ng/µL), 2.5 µL of 10 µM primers, 5 µL of 10 × *Ex Taq* buffer (20 mM Mg^2+^ plus), 4 µL of dNTP mixture (2.5 mM each), and *TaKaRa Ex Taq* polymerase (Takara Bio, Otsu, Japan). PCR reactions were carried out in a TaKaRa PCR Thermal Cycler Dice^®^ *Touch* with the following conditions: initially 95 °C (5 min), then followed by 25 cycles of 95 °C (30 s), 60 °C (1 min), and 72 °C (1 min). PCR mixtures were kept at 4 °C. To sequence the purified PCR amplicons, the reaction mixture (10 µL) containing 1.5 µL of ×5 sequence buffer (Thermo Fisher Scientific, Waltham, MA, USA), 1 µL of each primer, 1 µL of DNA template, 5.5 µL of sterile distilled milliQ water, and 1 µL of BigDye terminator™ v3.1 (Thermo Fisher Scientific) was performed in similar conditions as described above. After purification using the ethanol precipitation method, the amplicons were subjected to DNA sequencing using an ABI PRISM 3130*xl* Genetic Analyzer. Additionally, the shotgun metagenomic reads were mapped to the 16S rRNA gene sequences of representative isolates for the ARDRA-based OTUs, and then the relative abundances of the OTUs were calculated as gene copies per 1 million mapped reads (CPM), using the above-mentioned methods.

### 2.9. Measurement of the Potential Activity of Denitrification

The potential denitrification activities of each strain were determined using the acetylene-block method, as described previously [29,30]. Isolates were incubated in 5 mL of liquid DNB-NS medium in aerobic conditions for 3 weeks. After that, 30 µL of liquid culture (optical density measured at a wavelength of 600 nm was set at 0.7) was transferred into 20 mL vials containing 3 mL of liquid DNB-NS medium and covered by a rubber cap. The headspace air was replaced with dinitrogen (N_2_) gas using a Replace Injector GR-16 (Sanshin, Yokohama, Japan) followed by 2 mL Ar-C_2_H_2_ (90:10) gas injection into vials using a sterile 5 mL syringe. After incubation at 28 °C for 2 weeks, 1 mL of the headspace gas was transferred into a new 20 mL vial containing N_2_ gas. Thereafter, about 1 mL of each diluted sample was analyzed for N_2_O concentration using a gas chromatography GC-14A machine (electron capture detector-ECD type, Shimadzu). The quantity of water dissolved N_2_O was calculated as Bunsen absorption coefficient, then strains reducing ≥20% of added NO_3_^−^ to N_2_O were considered as denitrifying bacteria [37].

### 2.10. Phylogenetic Analysis

To assign the taxonomy of strains at the genus level, we filtered and trimmed the partial 16S rRNA gene sequences using seqtrace version 0.9.0 and searched the sequences using RDP seqmatch version 3 against RDP database (release11_6) [38]. All reference sequences and the related out sequences were aligned to construct a phylogenetic tree based on the maximum likelihood method using a Tamura 3-parameter model with 1000 bootstrap replicates within MEGA7 software (https://www.megasoftware.net, accessed on 24 November 2022) [39].

### 2.11. Accession Number

Metagenomic raw sequence data obtained in this study have been submitted to DDBJ/NCBI/EMBL under accession number DRA010653. The metagenome-assembled contigs, as well as the predicted CDSs with functional annotations and relative abundance have been deposited in the scientific repository Figshare with the DOI number 10.6084/m9.figshare.19328753. The partial 16S rRNA gene sequences obtained in the present study have been deposited in the DDBJ database with the following accession numbers: LC699492–LC699525.

## 3. Results

### 3.1. Chemical Properties of Substrates in Site IG7

In the 2016 investigation of site IG7, a new organic soil layer (the A layer) approximately 10 cm in thickness was observed, and in the 2018 investigation, the A layer buried the volcanic deposits (the C1 and C2 layers) (Figure 1D). The concentrations of total carbon and total nitrogen, as well as nitrate (NO_3_^−^) and ammonium (NH_4_^+^) in soil post-eruption, were approximately 3- to 40-fold higher than those in volcanic deposits that contained the least carbon and nitrogen sources (Table 1). In addition, these samples showed high carbon/nitrogen (C/N) ratios of 40.5 in 2017 and 19.8 in 2018, respectively (Table 1). The total carbon content in 2017 and 2018 had increased, which appeared to be related to the development of *Miscanthus*. Although there was no remarkable difference in NO_3_^−^ concentration on the surface at the IG7 site between March 2016 and September 2018, the concentration of NH_4_^+^ in September 2018 apparently increased compared with that in 2016. The least water content in volcanic deposits was also found, and all substrate samples were exposed to acidic conditions (Table 1).

### 3.2. Metagenome Assembly and Nitrogen Cycle-Related Gene Abundance

A total of 24.7 million high-quality paired-end reads (2 × 150 bp) was assembled into 1,194,015 contigs with a total length, maximum length, and *N_50_* value of 710,571,951 bp, 264,351 bp, and 569 bp, respectively. According to the taxonomic identification by Kraken, 541,517 bacterial, 4890 eukaryotic, 623 archaeal, and 381 viral contigs were identified for the *Miscanthus* rhizosphere microbiome, whereas the rest of the contigs were not successfully assigned to any known taxonomies. Furthermore, 1,499,158 CDSs were predicted from the metagenome assembly, and 71% of the predicted CDSs were assigned to COG categories. The most abundant categories with well-known functions were amino acid transport and metabolism (8%), carbohydrate transport and metabolism (5%), energy production and conversion (5%), cell wall/membrane/envelope biogenesis (4%), signal transduction mechanisms (4%), and replication/recombination/repair (3%).

The bacterial contigs were further classified to assess the taxonomic structure of the rhizosphere bacteriome of *Miscanthus*. A total of 76 bacterial taxa at the class level were identified in the *Miscanthus* rhizosphere. Gamma-proteobacteria (57%) was the most abundant taxon followed by the classes Alpha-proteobacteria (21%), Beta-proteobacteria (8%), Actinomycetia (7%), Acidobacteria (1%), Planctomycetia (0.5%), and Bacilli (0.2%). Further analysis of the class level showed the dominance of the families Enterobacteriaceae (26%), Erwiniaceae (10%), Pseudomonadaceae (9%), Aeromonadaceae (4%), and Xanthomonadaceae (1%) in the class Gamma-proteobacteria; the families Bradyrhizobiaceae (10%), Rhizobiaceae (1%), and Phyllobacteriaceae (1%) in the class Alpha-proteobacteria; the families Burkholderiaceae (6%), Comamonadaceae (1%), and Oxalobacteraceae (1%) in the class Beta-proteobacteria.

**Figure 2:**
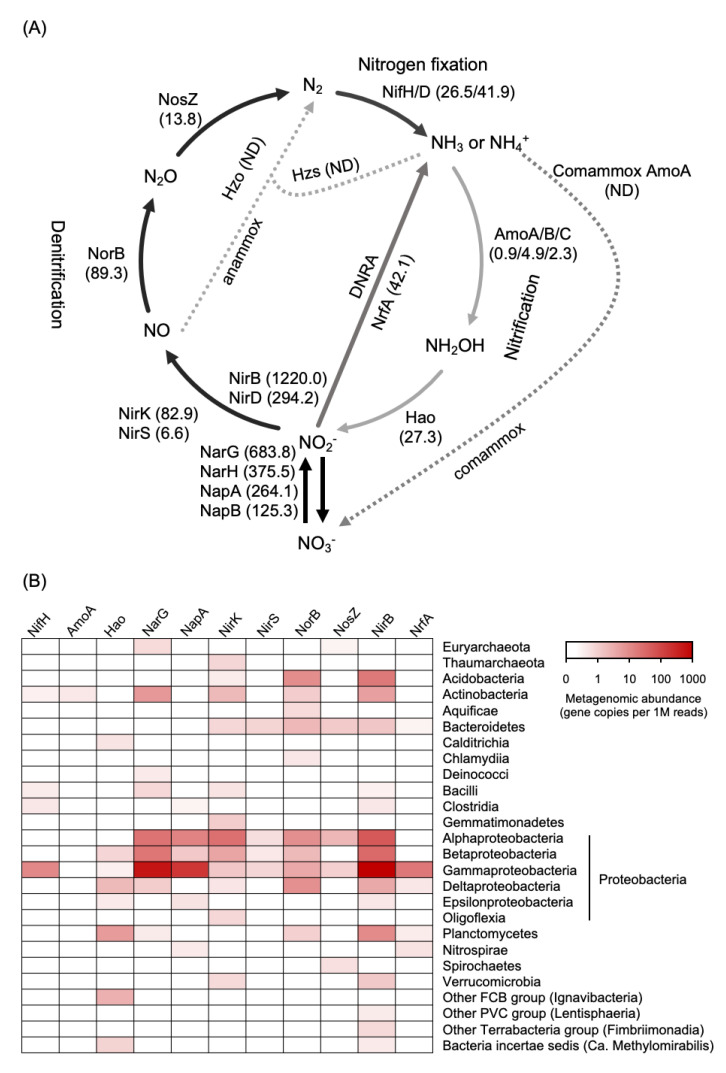
Nitrogen cycle-related genes based on identification with the KEGG database. (**A**), schematic of nitrogen-cycling processes. Arrows indicate direction of reaction. The numbers in parentheses represent the relative abundance of the genes described as gene copies per 1 million mapped reads (CPM). The key enzymes involved in the denitrification process (Nar, Nap, Nir, Nor, and Nos); nitrogen fixation (Nif); dissimilatory nitrate reduction to ammonium (DNRA; NrfA, NirB, and NirD); nitrification process (Hao, and Amo); assimilatory nitrate reduction (Nas); anaerobic ammonium oxidation (anammox; Hzo, and Hzs) are selected to show the nitrogen-cycling process. (**B**), heatmap shows taxonomic profile of the key genes involved in the nitrogen-cycling process.

DNA shotgun sequence analysis was carried out to identify genes that are directly influenced by root activities, preserved as the primary site for plant-microbe interaction, and tolerant of low nutrient conditions in the *Miscanthus* rhizosphere. We identified genes encoding the enzymes/proteins involved in chemotaxis and secretion systems, stress response sigma factors, transporters, and a mediator of the stringent response that coordinates a variety of cellular activities in response to changes in nutritional abundance (Table 2). On the other hand, nitrogen cycle-related genes encoding the fixation, reduction, and oxidation enzymes based on the KEGG nitrogen metabolic pathway comprised 0.11% (1662 CDSs) of the total predicted genes. Functional annotation of nitrogen cycle-related genes revealed highly consistent numbers for denitrification (*nar*, *nap*, *nir*, *nor*, and *nos*), nitrogen fixation (*nif*), respiratory DNRA (*nrfA*), and fermentative DNRA (*nirB* and *nirD*) (Figure 2A). Further analysis of the denitrifying bacterial community showed that *nirK* (68 CDSs) and *nirS* (6 CDSs) were mainly conserved in the genomes of members in the classes Alpha-, Beta- and Gamma-proteobacteria (Figure 2B). The specific key gene *nrfA* (19 CDSs) of the respiratory DNRA bacteria was mainly close to those possessed by Gamma-proteobacteria, whereas the gene *nirB* (547 CDSs) from fermentative DNRA bacteria was associated with a more diverse taxa including Acidobacteria, Planctomycete, Alpha-, Beta-, and Gamma-proteobacteria (Figure 2B). In addition, 19 CDSs encoding NifH (total abundance: 26.5 copies per 1 million reads) were detected and mainly identified in the class Gamma-proteobacteria (Figure 2B). Other nitrogen-cycling-related genes such as nitrification, assimilatory nitrate reduction to ammonium, anaerobic ammonium oxidation (anammox), and complete ammonia oxidation, were rarely found.

### 3.3. Diversity and Taxonomy of Cultured Bacteria with Potential Nitrogen Transformation in the Miscanthus Rhizosphere

The diversity of the culturable bacteria was determined by T-RFLP profiling using four different restriction enzymes. According to the T-RF profiles, the α-diversity index of each sample was not significantly different, suggesting less impact from different culture conditions as well as root types (Table 3). However, by adding an antibiotic, nalidixic acid in anaerobic conditions, each diversity index of culturable bacteria was slightly reduced compared with those indices in anaerobic conditions without the antibiotics (Table 3). In addition, the culturable bacterial communities in different culture conditions were grouped into two clusters (Supplementary Figure S2). One cluster contained those isolated from the rhizosphere of the main and lateral roots in aerobic conditions and from those of the main root in anaerobic condition with antibiotics, whereas the communities incubated in the other anaerobic conditions were clustered together.

**Figure 3:**
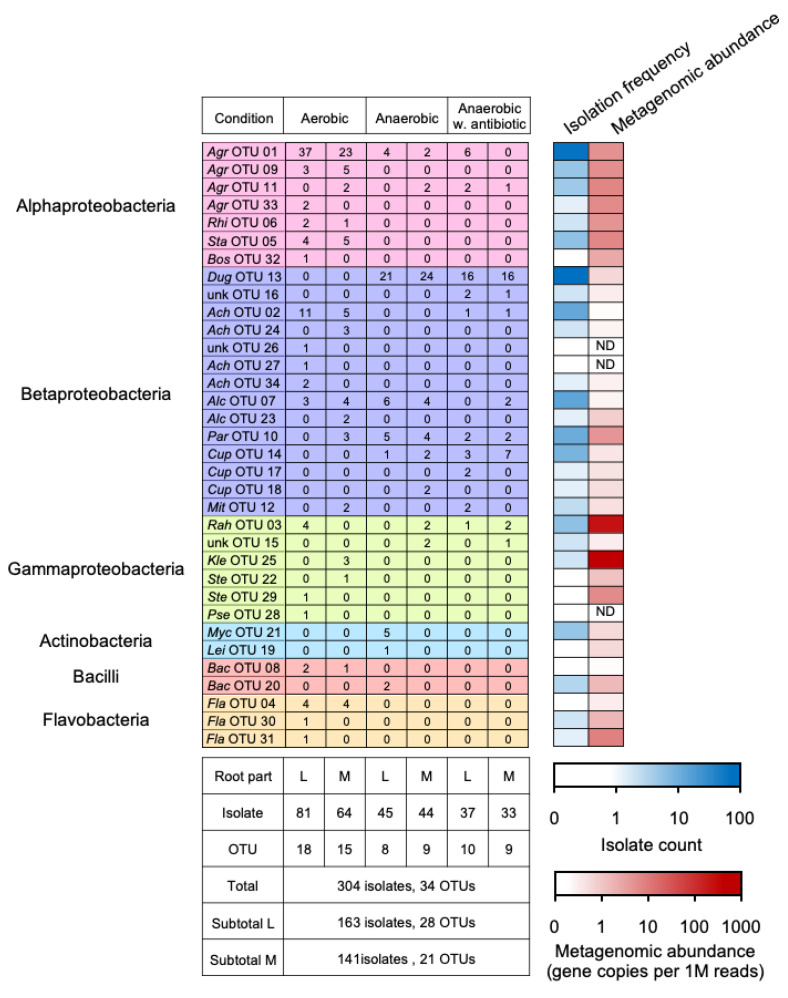
Bacterial isolates with potential denitrification functions from the *Miscanthus* rhizosphere. The upper-left table shows the distribution of OTUs, which were defined using amplified ribosomal DNA restriction analysis (ARDRA). The blue and red heatmaps show the isolate numbers and rhizospheric abundances of the OTUs, respectively. The lower-left table shows the numbers of bacterial isolates and OTUs using different isolation strategies. Abbreviations: *Agr*, *Agrobacterium*; *Rhi*, *Rhizobium*; *Sta*, *Starkeya*; *Bos*, *Bosea*; *Dug*, *Duganella*; *Ach*, *Achromobacter*; *Alc*, *Alcaligenes*; *Par*, *Paraburkholderia*; *Cup*, *Cupriavidus*; *Mit*, *Mitsuaria*; *Rah*, *Rahnella*; *Kle*, *Klebsiella*; *Ste*, *Stenotrophomonas*; *Pse*, *Pseudomonas*; *Myc*, *Mycobacterium*; *Lei*, *Leifsonia*; *Bac*, *Bacillus*; *Fla*, *Flavobacterium*; unk, unknown; L, lateral roots; M, main roots; ND, not detected.

A total of 304 isolates were obtained in six culture conditions and assigned to 34 OTUs according to the RF patterns of each strain generated by ARDRA, and a representative strain was randomly selected from each OTU to identify the taxonomy (Figure 3 and Table 4). Nucleotide sequences of 16S rRNA genes were determined and designated as “IG7-*n*”, where *n* is the OTU number (Table 4). In addition, the metagenomic reads were mapped to the 16S rRNA gene sequences to deduce the relative abundance of OTUs and/or strains in the rhizosphere community (Figure 3). These results showed that the isolated OTUs were affiliated to four phyla (Proteobacteria, Actinobacteria, Firmicutes, and Bacteroidetes), six classes (Alpha-proteobacteria, Beta-proteobacteria, Gamma-proteobacteria, Actinobacteria, Bacilli, and Flavobacteria), and eighteen genera (*Agrobacterium*, *Rhizobium*, *Starkeya*, *Bosea*, *Duganella*, *Achromobacter*, *Alcaligenes*, *Paraburkholderia*, *Cupriavidus*, *Mitsuaria*, *Rahnella*, *Klebsiella*, *Stenotrophomonas*, *Pseudomonas*, *Mycobacterium*, *Leifsonia*, *Bacillus*, and *Flavobacterium*) (Table 4 and Figure 3).

In summary, 7 OTUs (OTU_01, 02, 03, 07, 10, 11, and 12) were isolated in both aerobic and anaerobic conditions, whereas 18 OTUs (OTU_04, 05, 06, 08, 09, 22, 23, 24, 25, 26, 27, 28, 29, 30, 31, 32, 33, and 34) and 9 OTUs (OTU_13, 14, 15, 16, 17, 18, 19, 20, and 21) were isolated only in aerobic and anaerobic conditions, respectively (Figure 3). In addition, 6 OTUs (OTU_15, 18, 22, 23, 24, and 25) and 11 OTUs (OTU_19, 21, 26, 27, 28, 29, 30, 31, 32, 33, and 34) were isolated only from the main and lateral roots, respectively. OTU_01 (relative abundance, 24%) and OTU_13 (25%) were isolated as the most abundant OTUs in aerobic and anaerobic conditions and were affiliated with the genera *Agrobacterium* and *Duganella*, respectively (Table 4 and Figure 3). However, the related 16S rRNA gene sequences of the two OTUs were rarely detected in the rhizosphere community, which was revealed by shotgun metagenome. (Figure 3).

Mapping the metagenomic reads to the 16S rRNA gene sequences of the isolated strains revealed that the most abundant OTU harbored in the rhizosphere was OTU_25 (Genus, *Klebsiella*), followed by OTU_3 (*Rahnella*), 20 (*Leifsonia*), 11 (*Agrobacterium*), 5 (*Starkeya*), 33 (*Agrobacterium*), 29 (*Stenotrophomonas*), 9 (*Agrobacterium*), 1 (*Agrobacterium*), 6 (*Rhizobium*), and 10 (*Paraburkholderia*), suggesting their dominance in the *Miscanthus* rhizosphere, whereas the other strains were characterized as minorities with relative abundances lower than 10.0 CPM (Figure 3). Additionally, strains OTU_26, 27, and 28 were not detected (<0.04 CPM) in the metagenome dataset (Figure 3).

### 3.4. Denitrifying Activity

**Figure 4:**
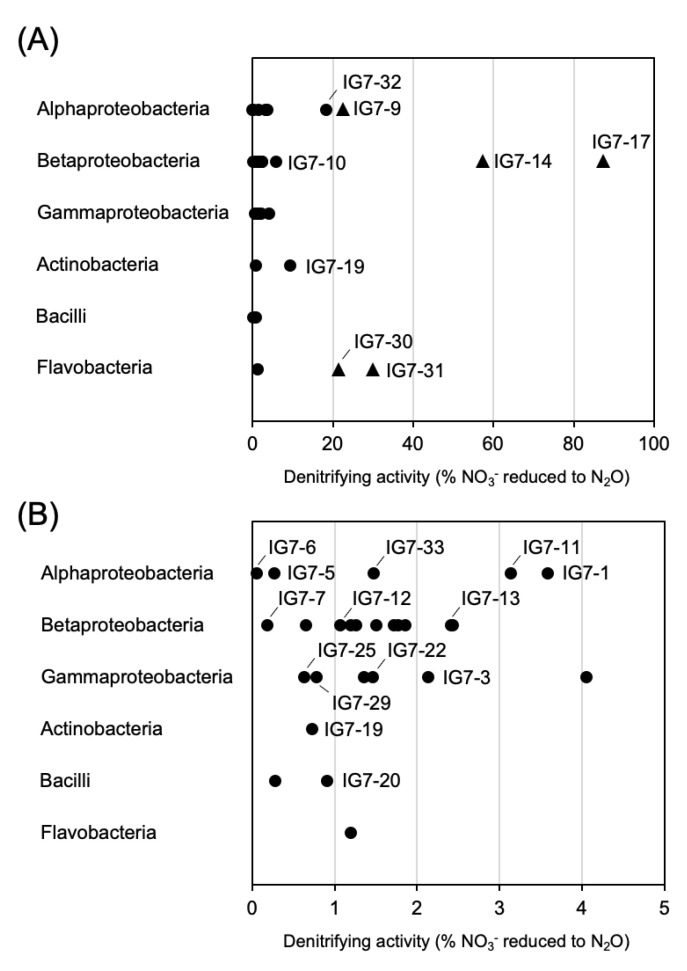
The denitrifying activity of OTUs. (**A**) shows strains that reduced more than 5% of the supplied NO_3_^−^ to N_2_O. Black circles and triangles indicate strains that produced N_2_O from the supplied NO_3_^−^ at levels of <20% and >20%, respectively. (**B**) shows strains that reduced less than 5% of the supplied NO_3_^−^ to N_2_O. The *x*-axis and *y*-axis show the denitrifying activity (%) and the class of OTUs, respectively.

In total, 34 representative strains of the ARDRA-based OTUs were determined for their potential denitrifying activity using the acetylene block method [29]. Five representative strains showed denitrifying activity that reduced more than 20% of the supplied nitrate to nitrous oxide (Figure 4A), and these strains were characterized as the denitrifying bacteria in accordance with Tiedje (1994) [37]. Strains IG7-14 and IG7-17 identified as the genus *Cupriavidus* within the class Beta-proteobacteria (Figure S3A), which were isolated from the main roots in anaerobic conditions and reduced 57.3% and 87.4% of the supplied nitrate to nitrous oxide, respectively (Figure 4). Strain IG7-9, identified as the genus *Agrobacterium* within the class Alpha-proteobacteria (Figure S3B), and strains IG7-30 and IG7-31 affiliated with the genus *Flavobacterium* within the class Flavobacteria (Figure S3C), which were obtained in aerobic conditions, were found to have reduced 20–30% of the suppled nitrate to nitrous oxide (Figure 4A). Representative strains affiliated with the classes of Gamma-proteobacteria, Actinobacteria, and Bacilli were able to reduce less than 9.4% of the suppled nitrite to nitrous oxide (Figure 4).

## 4. Discussion

Previous studies have revealed the role of *Miscanthus* as pioneer plants in disturbed and acidic soil as well as their ability to form root-symbiotic relationships with diazotrophic bacteria and arbuscular mycorrhizal fungi to promote their growth in unfavorable environments [10,21,40,41]. Guo et al. (2021) demonstrated that a dynamic succession occurred in the rhizosphere microbiome of *M. condensatus* across the developmental phases on recent Miyake-jima volcanic deposits [8]. Moreover, a unique community structure was determined for the *Miscanthus* rhizosphere forming on the Miyake-jima volcanic deposits [8]. This community structure was obviously different from the early soil microbial communities in the recent Miyake-jima volcanic deposits, as well as from the rhizosphere communities of the *Miscanthus* grass colonizing various extreme habitats in Taiwan. The former was dominated by Beta-proteobacteria [6], whereas the latter mainly consisted of the phyla Acidobacteria and Chloroflexi [42]. Considering the extremely low nitrogen content of the volcanic substrate (TN ≤ 0.2 g/kg dry sample in 2016–2018), the present study focused on the nitrogen-cycle driven by root-associated microorganisms when this pioneer plant developed on the volcanic substrate.

Total carbon and nitrogen contents in the soil increased three to seven times from 2016 to 2018 (Table 1), appearing to be simultaneous with the rapid development of *M. condensatus* in the same period. Rapid increases in soil organic matter may promote nitrate reductive processes conducted by heterotrophic microorganisms [43,44], and we found that ammonium content was two- to six-fold higher than that of NO_3_^−^ in all substrates in 2018, which was contrary to the tendency in 2016 (i.e., NO_3_^−^ > NH_4_^+^, Table 1). These results implied that nitrate reductive processes, i.e., the respiratory denitrification and DNRA, might occupy a major part of nitrogen cycling in the *M. condensatus*-colonized volcanic substrate, particularly where present in the rhizosphere.

In the metagenomic analysis, we identified genes encoding chemotaxis and Type III/IV/VI secretion systems, RpoN that mediates gene transcription involved in nitrogen metabolism and other cellular processes [45,46], bacterial ammonium transporters, and the *spoT*/*relA* homologue gene encoding eubacterial ppGpp. The latter is a mediator of the stringent response that coordinates a variety of cellular activities in response to changes in nutritional abundance (Table 2). These findings suggested that the rhizosphere is the narrow region of soil directly influenced by root activities, preserved as the primary site for plant-microbe interactions, and adapted to low nutrient conditions [47]. In addition, the plant roots actively secreted specific compounds that altered the bacterial community around the rhizosphere [48]. Thus, a selective association between plants and the rhizosphere microbiome will determine their ability to adapt and survive in harsh conditions [49].

With respect to the nitrogen cycle, our metagenomic data revealed that the key genes associated with denitrification (i.e., *nirK* and *norB*), respiratory DNRA (i.e., *nrfA*), and fermentative DNRA (i.e., *nirB* and *nirD*) were more abundant than those associated with nitrogen fixation (i.e., *nifH* and *nifD*) and nitrification (i.e., *amoA*, *amoB*, *amoC*, and *hao*) (Figure 2), which clearly illustrated the dominance of nitrate-reducing microbial community in the pioneer plant’s rhizosphere.

Respiratory denitrification is characterized by the formation of N_2_ and/or nitrous oxide (N_2_O) gas as a product of NO_3_^−^ or NO_2_^−^ reduction, followed by a greater increase in strain growth compared with if nitrogen oxide served as a terminal electron acceptor [37]. Our metagenomic results also showed that *nirK* and *nirS* were mainly affiliated with the classes Alpha-, Beta- and Gamma-proteobacteria (Figure 2). Anaerobically isolated strains IG7-14 (i.e., OTU14) and IG7-17 (i.e., OTU17), which belonged to the genus *Cupriavidus* (Beta-proteobacteria), showed higher in vitro denitrifying activity than other cultured strains (Figure 4); however, their 16S rRNA gene abundances were low in the rhizosphere (Figure 3). On the contrary, the other strains with low in vitro denitrifying activity seemed to dominate in the rhizosphere, which included members in the classes Flavobacteria, Alpha-proteobacteria, and Gamma-proteobacteria (Figure 3 and Figure 4).

DNRA activity is mainly performed through an anaerobic microbial pathway, but Morley and Baggs (2010) demonstrated that it occurs even when O_2_ is as high as 21% (*v*/*v*) [50]. This process retains the nitrogen source in the form of NH_4_^+^ [51,52], which serves as N-nutrition for primary producers and heterotrophic microbes [12]. In the present study, a large number of *nirB* and *nirD* genes encoding the large and small subunits of cytoplasmic NADH-dependent nitrite reductase, respectively, were detected in the *Miscanthus* rhizosphere (Figure 2), suggesting that fermentative DNRA bacteria occupied the nitrogen-reducing community associated with the rhizosphere. In addition to the fermentative DNRA-related genes, a considerable proportion of *nrfA* genes responsible for respiratory DNRA, which is closely related to those possessed by Gamma-proteobacteria, was also found in the rhizosphere (Figure 2).

Several studies have compared the contribution of DNRA to the total NO_3_^−^ consumption in biomes such as temperate and subtropical forests as well as temperate grasslands, which range from negligible to dominant, indicating the regulation of rates and the importance of DNRA by various environmental factors such as redox potential, C/N ratio, quality of carbon, NO_2_^−^/NO_3_^−^ ratio, sulfur, and iron concentrations [12,53]. Recently, DNRA activity in a soil ecosystem was proposed to be increased or may outcompete denitrification by high carbon availability, and NO_3_^−^ limitation is often expressed as the molar carbon/NO_3_^−^ ratio above 12 [54,55], and such conditions occurred very often in the rhizosphere [56]. Moreover, DNRA rates were affected by the type of plant root exudate (mainly consisting of organic acid) following the order oxalate > citrate > glucose > acetate > malate [57]. In addition, genomic analyses have shown that some Beta-proteobacteria denitrifiers also possess the *nirBDC*–*cysC* gene cluster of fermentative DNRA in their genome [58,59], which are thought to function in ammonification and detoxification of NO_2_^−^ when a huge amount of NO_3_^−^ is respirated to NO_2_^−^ by Nar [60]. Thus, the specific adaptive features of plants, as well as the active bacterial role in DNRA and denitrification, might be affected by the nutrient source type and availability around the *Miscanthus* rhizosphere [61,62].

A meta-analysis identified the classes of Gamma-proteobacteria and Alpha-proteobacteria consisting of the orders of Rhodobacterales, Rhodospirillales, Aeromonadales, and Alteromonadales to be the most important bacterium performing DNRA [12]. Our results confirmed that a large amount of some members belonging to these taxa inhabited the *Miscanthus* rhizosphere. In respect to the poor soil fertility and the heavy amount of annual precipitation on Miyake-jima, the aggressive association with DNRA bacteria in the rhizosphere may contribute toward the growth of *Miscanthus* on barren volcanic deposits by retaining nitrogen in the rhizosphere. For this reason, it could be suggested that the grass *M. condensatus* on the Miyake-jima volcanic deposits showed a specific pattern to shape a beneficial rhizobacterial community during their colonization.

Conversely, the negligible numbers of nitrification- and anammox-related genes in the current study might be explained by two reasons: (1) nitrification in acidic soil might be primarily performed by the fungal community [63] and other unsolved heterotrophic nitrifiers [64]; (2) the contribution of anammox in terrestrial ecosystems is usually low because of their specific ecological requirements [65,66,67,68]. Indeed, a few of the fungal contigs associated with the genera of *Ilyonectria*, *Parastagonospora*, *Aspergillus*, *Nectria*, *Diplodia*, *Cordyceps*, *Acremonium*, *Kwoniella*, and *Rhodotorula* have been detected in the metagenome dataset of the *Miscanthus* rhizosphere (data not shown).

## 5. Conclusions

In conclusion, the present study investigated the nitrogen-nutrient state of recent Miyake-jima volcanic deposits that were fully vegetated by the pioneer plant *M. condensatus* and evaluated the nitrogen-cycling microorganisms inhabiting the rhizosphere using a combination of shotgun metagenomics and culture-based analyses. Our results showed that nitrogen nutrients in the volcanic deposit increased with the development of the *Miscanthus*-dominated vegetation, and a unique nitrate-transforming bacterial community consisting of denitrifiers and DNRA-bacteria inhabiting the rhizosphere. Additionally, diverse bacteria encompassing three phyla (Proteobacteria, Actinobacteria, and Bacteroidetes) were isolated from the *Miscanthus*-rhizosphere through stimulation using nitrate and succinate. Those affiliated with the genus *Cupriavidus* within the class Beta-proteobacteria showed the highest in vitro denitrifying activity; however, the shotgun metagenomic results indicated that the DNRA-related rhizobacteria appeared to take a major role in the nitrogen cycle in the rhizosphere of *Miscanthus* pioneering on recent Miyake-jima volcanic deposits.

Metagenomics analysis results provide important evidence in the initiation of ecosystem development in Miyake-jima primary volcanic deposits. Moreover, this study increases understanding of the association between the *Miscanthus* rhizosphere and a nitrate-transforming bacterial community on the newly placed volcanic deposits. Further studies through the metatranscriptomic and/or metaproteomic approaches as well as metabolic stable isotope labelled nitrogen are needed to determine the exact nitrogen-cycle processes that are actively present during *Miscanthus* colonization.

## Figures and Tables

**Table 1:** Soil chemical properties of soil and volcanic deposit layer on site IG7.

Sampling Date/Layer		Soil Chemical Properties					
	TC g/kg Dry Soil	TN g/kg Dry Soil	C/N Ratio	NO_3_^−^ g/kg Dry Soil	NH_4_^+^ g/kg Dry Soil	pH (H_2_O)	Water Content (%)
Mar. 2016							
A layer	n.a.	n.a.	n.a.	n.a.	n.a.	n.a.	n.a.
C layer	0.4	0.1	4.0	0.02	<0.01	5.1	25.1
Sep. 2017							
A layer	8.1	0.2	40.5	n.d.	n.d.	5.0	19.5
C layer	0.2	N.D.	n.a.	n.d.	n.d.	4.9	10.9
Sep. 2018							
A layer	27.7	1.4	19.8	0.01	0.06	5.0	31.2
C1 layer	1.2	0.2	6.0	<0.01	0.02	4.9	11.8
C2 layer	0.6	0.1	6.0	<0.01	0.01	4.7	9.7

The measurement was performed three times and the average value was showed. A, C1, and C2 layers indicate the soil post-eruption, latter volcanic deposits, and former volcanic, respectively. n.a., not available; n.d., No data; N.D., not detected.

**Table 2:** Prediction-based number of selected key function and genes in *Miscanthus* rhizosphere soil of site IG7.

Selected Key Function * and Protein	Gene Name	Gene Count	Gene Copies per 1M Reads
DNA replication and recombination			
DNA gyrase	*gyrA* ^†^	738	122.6
	*gyrB* ^†^	721	148.1
DNA polymerase III subunit	*dnaN* ^†^	370	68.1
Recombinase	*recA* ^†^	372	66.2
Transcription			
DNA-directed RNA polymerase, alpha subunit	*rpoA* ^†^	306	104.4
DNA-directed RNA polymerase, beta subunit	*rpoB* ^†^	1000	218.7
DNA-directed RNA polymerase, beta subunit	*rpoC* ^†^	1036	277.0
DNA-directed RNA polymerase, omega subunit	*rpoZ*	120	36.2
RNA polymerase primary sigma factor	*rpoD* ^†^	793	131.8
RNA polymerase sigma-54 factor	*rpoN* (*ntrA*)	380	74.4
Extracytoplasmic function (ECF) sigma factors	*rpoE*	1256	160.4
Cell division			
Transpeptidase	*ftsI* ^†^	538	120.6
GTPase	*ftsZ* ^†^	368	68.1
Two-component regulatory system			
Chemotaxis protein, methyltransferase	*cheR*	298	54.5
Purine-binding chemotaxis protein	*cheW*	242	41.3
Chemotaxis protein	*cheX*	36	4.1
Response regulator	*cheY*	281	42.1
Secretion system			
Type III	*yscV* (*escV*)	49	8.0
Type IV	*virB4*	155	21.8
	*virD4*	215	22.7
Type VI	*vgrG*	905	183.9
	*hcp*	385	97.7
	*icmF* (*vasK*)	784	124.5
Stringent response			
ppGpp synthase/hydrolase	*spoT* (*relA*)	545	104.5
Transporter			
Ammonium transporter superfamily	*amt*	640	92.8

* The selected genes were used as markers for genes involved in each function. † Single copy genes in relation to housekeeping are showed.

**Table 3:** Diversity indices of culturable bacterial T-RFs in each culture conditions.

Culture Conditions	Samples	Diversity Index *		
		T-RF Types	*E*	*H*’
Aerobic	Lateral roots	19.5 ± 5.1	0.70 ± 0.09	2.06 ± 0.43
	Main roots	21.5 ± 3.1	0.82 ± 0.04	2.50 ± 0.23
Anaerobic	Lateral roots	14.0 ± 3.4	0.79 ± 0.05	2.08 ± 0.30
	Main roots	14.5 ± 2.4	0.76 ± 0.05	2.01 ± 0.16
Anaerobic with antibiotic	Lateral roots	13.3 ± 2.6	0.76 ± 0.06	1.95 ± 0.28
	Main roots	13.8 ± 2.9	0.76 ± 0.06	1.98 ± 0.31

* The filtered T-RF data abundance outputted from T-REX software was examined for richness (T-RF types), evenness (*E*), and Shannon’s index (*H*’), respectively.

**Table 4:** Taxonomic identification of representative strain for the ARDRA-based OTUs.

OTUs	Strain Name	Phylogenetic Relationship		
		Closest Organism	Accession no.	S_ab Score *
01	IG7-1	*Agrobacterium radiobacter*	AB247582	0.995
02	IG7-2	*Achromobacter* sp.	HQ256543	0.555
03	IG7-3	*Rahnella aquatilis*	KJ781940	0.993
04	IG7-4	*Flavobacterium johnsoniae*	AB681010	0.984
05	IG7-5	*Starkeya* sp.	KC904964	0.958
06	IG7-6	*Rhizobium* sp.	KF465952	0.955
07	IG7-7	*Alcaligenes faecalis*	AJ509012	0.990
08	IG7-8	*Bacillus cereus*	CM000739	0.994
09	IG7-9	*Agrobacterium radiobacter*	AB247582	0.968
10	IG7-10	*Paraburkholderia unamae*	KM974658	0.967
11	IG7-11	*Agrobacterium radiobacter*	AB247582	0.993
12	IG7-12	*Mitsuaria chitosanitabida*	AB006851	0.990
13	IG7-13	*Duganella* sp.	HQ829837	0.917
14	IG7-14	*Cupriavidus* sp.	JN128831	0.972
15	IG7-15	*Enterobacteriaceae* bacterium	CP003938	0.511
16	IG7-16	*Oxalobacteraceae* bacterium	DQ337591	0.984
17	IG7-17	*Cupriavidus* sp.	JN226398	0.990
18	IG7-18	*Cupriavidus* sp.	HE662654	0.969
19	IG7-19	*Leifsonia* sp.	KT462730	0.794
20	IG7-20	*Bacillus cereus*	JN579711	0.920
21	IG7-21	*Mycobacterium* sp.	AY188086	1.000
22	IG7-22	*Stenotrophomonas maltophilia*	KR007965	0.840
23	IG7-23	*Alcaligenes faecalis*	AJ509012	0.985
24	IG7-24	*Achromobacter denitrificans*	FJ810080	0.990
25	IG7-25	*Klebsiella* sp.	HQ204298	0.973
26	IG7-26	Beta-proteobacterium	AB607287	0.713
27	IG7-27	*Achromobacter xylosoxidans*	JN381516	0.507
28	IG7-28	*Pseudomonas mendocina*	KP324955	0.365
29	IG7-29	*Stenotrophomonas maltophilia*	GQ360071	0.993
30	IG7-30	*Flavobacterium johnsoniae*	AB681010	0.974
31	IG7-31	*Flavobacterium johnsoniae*	AB078043	0.848
32	IG7-32	*Bosea* sp.	AB542375	0.988
33	IG7-33	*Agrobacterium* sp.	KF465838	0.985
34	IG7-34	*Achromobacter* sp.	KF059272	0.979

* The S_ab score is the number of (unique) seven-base oligomers shared between the query sequence and a given RDP sequence divided by the lowest number of unique oligos in either of the two sequences [38].

## Data Availability

Metagenomic raw sequence data obtained in this study have been submitted to DDBJ/NCBI/EMBL under the accession number DRA010653. The metagenome-assembled contigs, as well as the predicted CDSs with functional annotations and relative abundance have been deposited in the scientific repository Figshare with the DOI number 10.6084/m9.figshare.19328753. The partial 16S rRNA gene sequences obtained in the present study have been deposited in the DDBJ database with the following accession numbers: LC699492–LC699525.

## Supplementary Materials

The following supporting information can be downloaded at: https://www.mdpi.com/article/10.3390/microorganisms11020260/s1, Figure S1: Pictures shown the roots of Miscanthus collected from Miyake-jima in 2018, Figure S2: Dendogram based on T-RFLP profiles shows the similarity among the culturable communities in different culture conditions, Figure S3: Maximum likelihood tree based on 16S rRNA gene sequences int the class Betaproteobacteria (Tamura 3-parameter model) (A), class Alphaproteobacteria (Tamura 3-parameter model) (B), and genus *Flavobacterium* within the class Flavobacteria (kimura 2-parameter model) (C).
