# Supplementary material for: A Nitrate-Transforming Bacterial Community Dominates in the Miscanthus Rhizosphere on Nitrogen-Deficient Volcanic Deposits of Miyake-jima
Source: Microorganisms. 2023 Jan 19;11(2):260. doi: 10.3390/microorganisms11020260 (PMC9961740; doi:10.3390/microorganisms11020260)
Supplement: Supplementary file 1 [file microorganisms-11-00260-s001.zip › microorganisms-2100912-supplementary.pdf]

**Supplementary Figure S1.**

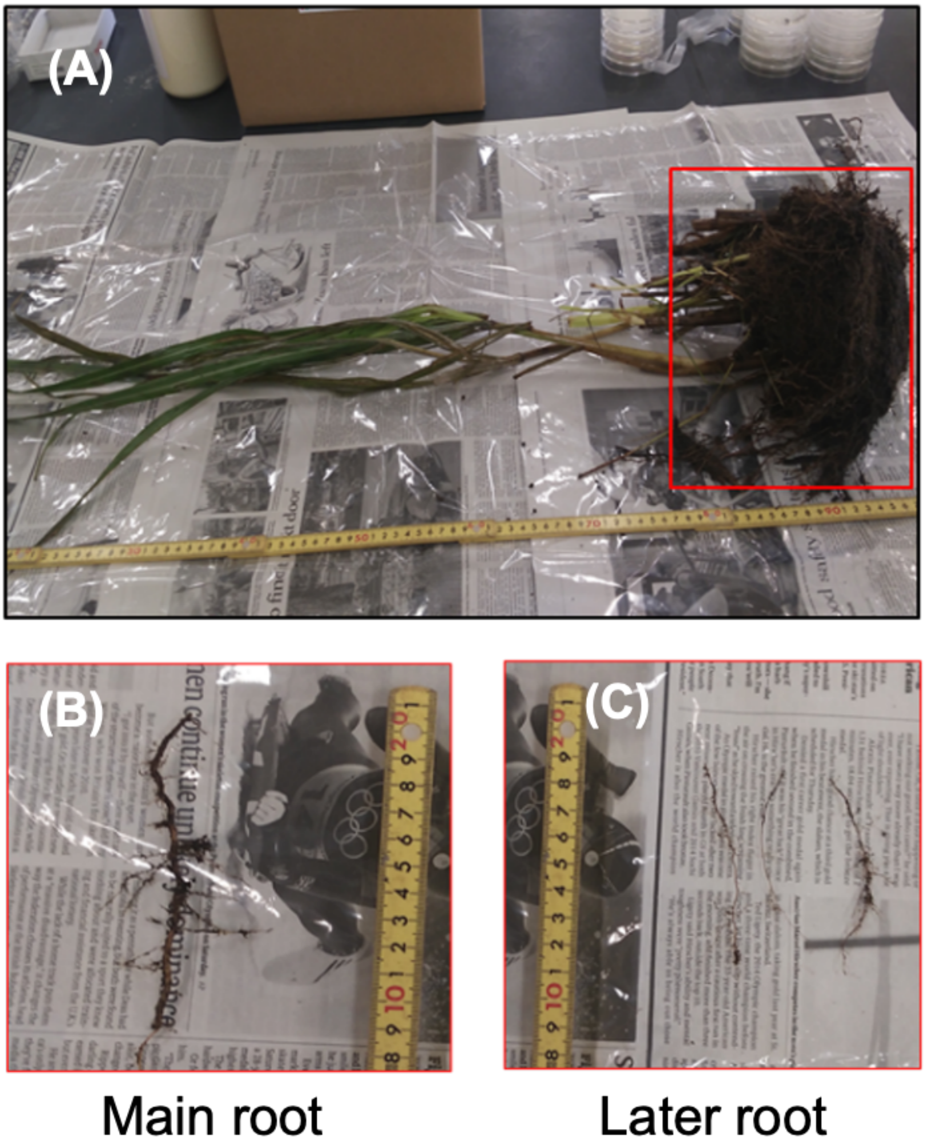


**Supplementary Figure S1.** Pictures shown the roots of *Miscanthus* collected from Miyake-jima in 2018. (A) *Miscanthus* plant with rhizosphere soil; (B) Main root; (C) Later root.

**Supplementary Figure S2.**

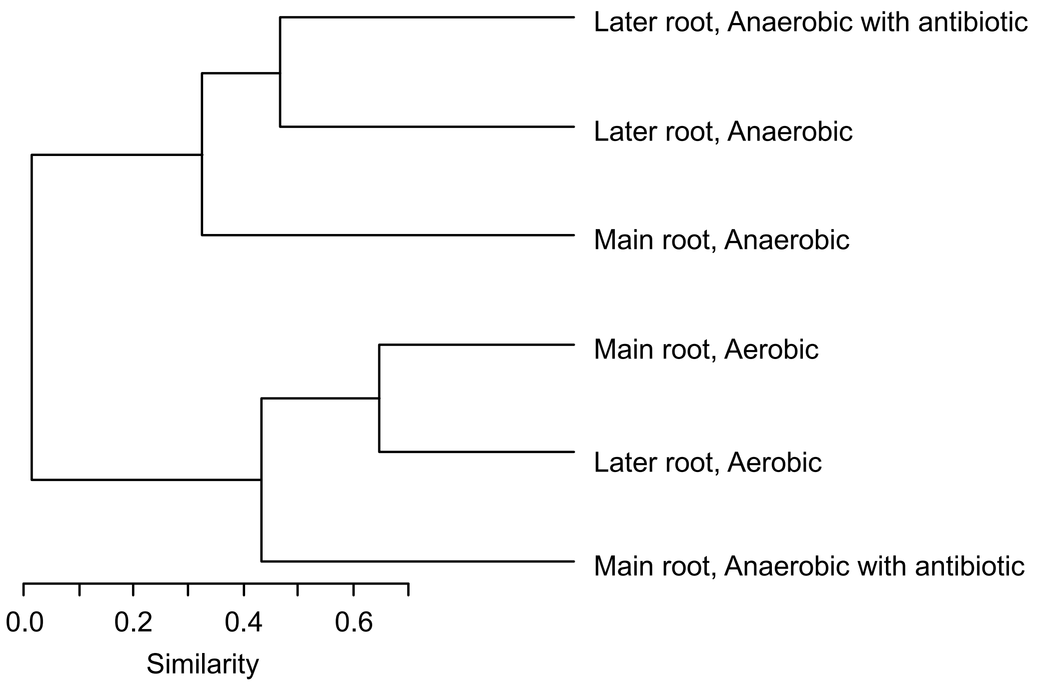


**Supplementary Figure S2.** Dendogram based on T-RFLP profiles shows the similarity among the culturable communities in different culture conditions.

## Supplementary Figure S3.

(A)

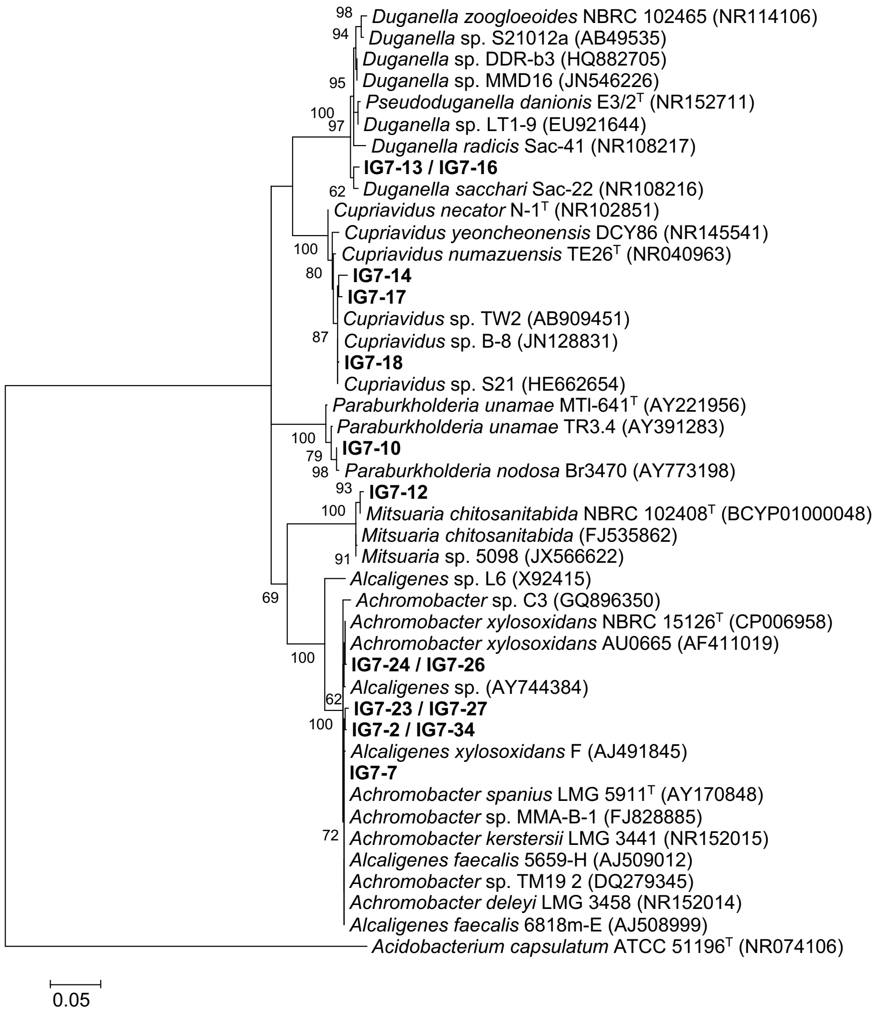

**Supplementary Figure S3.**

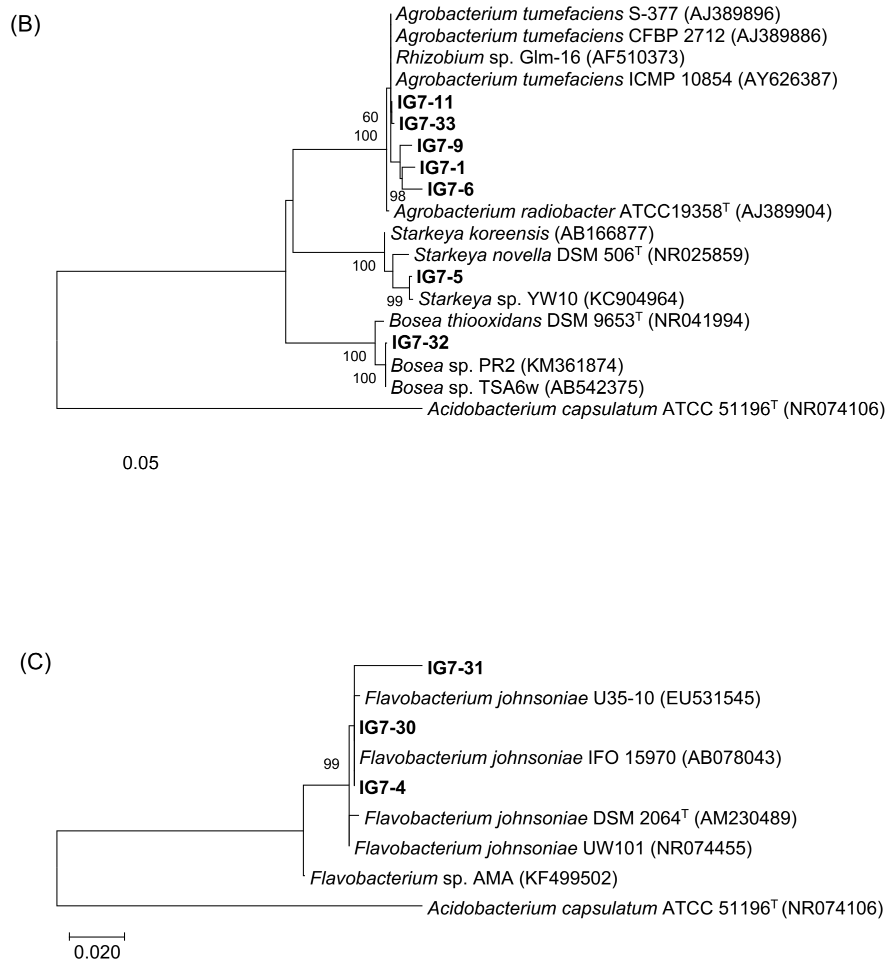


**Supplementary Figure S3.** Maximum likelihood tree based on 16S rRNA gene sequences in the class Betaproteobacteria (Tamura 3-parameter model) (A), class Alphaproteobacteria (Tamura 3-parameter model) (B), and genus *Flavobacterium* within the class *Flavobacteria* (Kimura 2-parameter model) (C). The 16S rRNA gene sequence of *Acidobacterium capsulatum* ATCC 51196$^{T}$ was used as an outgroup. Values more than 60% are indicated at branches with 1,000 bootstrap replicates.
